# Trends towards Biomimicry in Theranostics

**DOI:** 10.3390/nano8090637

**Published:** 2018-08-21

**Authors:** Michael Evangelopoulos, Alessandro Parodi, Jonathan O. Martinez, Ennio Tasciotti

**Affiliations:** 1 Center for Biomimetic Medicine, Houston Methodist Research Institute, Houston, TX 77030, USA; mevangelopoulos@houstonmethodist.org (M.E.); jomartinez@houstonmethodist.org (J.O.M.); 2 Department of Pharmacology, University of Illinois at Chicago, Chicago, IL 60607, USA; aparodi@uic.edu; 3 Department of Orthopedics & Sports Medicine, Houston Methodist Hospital, Houston, TX 77030, USA

**Keywords:** biomimetic, bioinspired, cancer, multistage nanovectors, nanomedicine, nanoparticles, theranostics

## Abstract

Over the years, imaging and therapeutic modalities have seen considerable progress as a result of advances in nanotechnology. Theranostics, or the marrying of diagnostics and therapy, has increasingly been employing nano-based approaches to treat cancer. While first-generation nanoparticles offered considerable promise in the imaging and treatment of cancer, toxicity and non-specific distribution hindered their true potential. More recently, multistage nanovectors have been strategically designed to shield and carry a payload to its intended site. However, detection by the immune system and sequestration by filtration organs (i.e., liver and spleen) remains a major obstacle. In an effort to circumvent these biological barriers, recent trends have taken inspiration from biology. These bioinspired approaches often involve the use of biologically-derived cellular components in the design and fabrication of biomimetic nanoparticles. In this review, we provide insight into early nanoparticles and how they have steadily evolved to include bioinspired approaches to increase their theranostic potential.

## 1. Introduction

Over the past several decades, medicine has benefitted significantly from the use of imaging modalities to help guide diagnosis and treatment. While our ability to look inside the body was initially largely limited to what could be felt, the introduction of more advanced imaging systems (e.g., X-ray imaging) helped revolutionize the field of imaging and is now among medicine’s leading diagnostic tools. Since then, imaging modalities to treat diseases have evolved from simple X-rays to high resolution computer augmented virtual environments that allow physicians to navigate the various layers of the body in greater detail [1,2,3,4]. However, despite imaging systems evolving to generate great detail and delineate the complexity of the body, diagnosis and treatment algorithms continue to remain a two-step process, consequently limiting the onset of therapy [5]. More so, although nanotechnology has been introduced as an effective utility to concentrate a payload to a target site [6,7,8], thereby limiting toxicity to healthy tissue and other side effects, this approach continues to require two distinct steps to diagnose and treat disease. To mitigate these shortcomings, significant interest has been sparked towards the development of therapies that aim to combine diagnostic and therapeutic capabilities into a single agent.

This new class of treatment, referred to as theranostics, has led to the development of a large arsenal of therapeutic agents that offer a viable one-step treatment solution [9,10,11]. For example, nanomaterials capable of enhancing tumor imaging while concurrently delivering a therapy is only one application in which theranostic-based technologies are being exploited [12,13]. With the urgency required in the timely diagnosis and subsequent treatment of cancer, time-saving theranostic treatments have garnered tremendous support [5,14,15,16].

Recently, in an effort to further strengthen the effectiveness of theranostics, bioinspired approaches have been developed with a goal of providing biological-like behaviors to synthetic theranostic vectors. In this review, we outline the fundamental imaging modalities that have largely contributed to the development of theranostic-based therapies followed with a discussion on multi-step delivery vectors that have contributed to furthering efficacy for these imaging modalities. Lastly, a brief overview of bioinspired theranostic strategies is discussed.

## 2. Nanoparticle-Based Theranostics

### 2.1. Iron Oxide Nanoparticles

Iron Oxide nanoparticles (IONP) have generated tremendous momentum in nanomedicine due to their many beneficial properties [17]. Distinctive elements such as superparamagnetism, susceptibility to surface-modifications (e.g., polyethylene glycol, dextran, polypeptides, etc.), and high surface to volume ratios have proven highly useful, particularly for magnetic resonance imaging (MRI) and drug delivery [18,19,20]. Composed of ferrite nanocrystallites of magnetite and their oxidized counterpart maghemite, the last decade has witnessed considerable interest in these particles for theranostic applications. Specifically, it has been found that when IONP are reduced to a size of <20 nm, they become superparamagnetic in the presence of a magnetic field [21]. Conversely, when the magnetic field is turned off, the particles become highly dispersed [22]. In clinical applications, this feature is critical as the aggregation of particles can lead to detection and sequestration by the mononuclear phagocyte system (MPS), inhibiting IONP from reaching their target and significantly lowering their efficacy [23].

**Figure 1:**
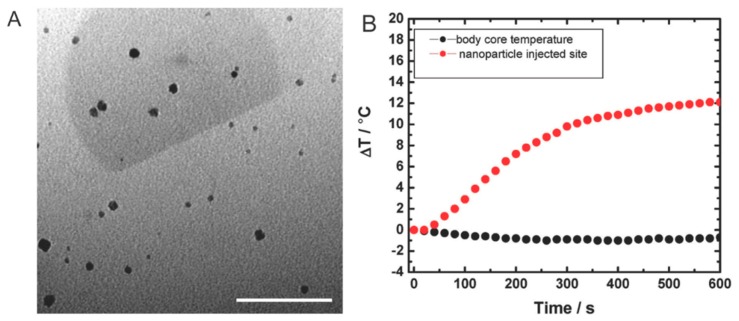
(**A**) Transmission electron microscope image of IONP fabricated with a dopamine-anchored shell, scale bar, 100 nm; (**B**) Quantitative analysis depicted the temperature changes at the nanoparticle injection site versus the body core as measured with a fiber optic temperature probe. Images reproduced from [25], with permission from BioMed Central Ltd., 2010.

These features, coupled with the use of a magnetic field as a guiding mechanism, can be beneficial in a number of ways. For example, the total drug amount needed to achieve a clinical effect can be reduced, resulting in a decrease in the frequency of administration and minimal cytotoxic effects on healthy tissue [24]. Furthermore, when subjected to an alternating magnetic field, IONP have also been shown to dissipate heat, resulting in an increase in temperature in the surrounding area. This feature has been exploited in magnetic hyperthermia to kill cancer cells, resulting in an increase of over 10 °C at the injection site (Figure 1) [25]. Meanwhile, surface modifications (e.g., antibodies, dyes, chemotherapeutics) garnered beneficial properties for IONP by prolonging circulation [26] and increasing cancer-targeting abilities [27]. In one case, IONP were functionalized with cystine, the oxidized dimer of cysteine, to achieve improved biocompatibility and hydrophilicity [28]. In addition, cystine-functionalized IONP demonstrated versatility as a viable contrast agent for MRI, as well as ultrasonography, further exhibiting its potential for theranostic applications.

While multifunctional nanoparticles have recently gained significant attention, improvements still need to be made in the loading ability of nanoparticles into the drug carrier. Yoon et al. reported that IONP co-loaded with the chemotherapeutic, paclitaxel, into micelles, showed promising results as a candidate for the combined imaging and treatment of cancer [29]. These results show micelles, encapsulated with both a chemotherapeutic payload and imaging agent, were able to inhibit the growth of a tumor in vivo by more than 50% compared to a control group, thereby demonstrating this coupled approach as a promising theranostic tool. Nevertheless, limitations of IONP continue to exist. For example, the physiological environment of the body causes drug-conjugated IONP to suddenly release the payload upon administration, thereby limiting its effectiveness at the intended site. Efforts to mitigate the adverse release of payloads have been achieved through the incorporation of layer-by-layer fabrication using oppositely charged polymers [30]. This attempt exhibited the development of a stabilized IONP formulation while also achieving the simultaneous loading of naturally-derived compounds.

In general, multi-modal systems incorporating IONP have drawn considerable attention for their magnetic and photothermal properties. In more recent efforts, merging polymer responsive materials with IONP have exhibited desirable properties through the manipulation of environmental factors to achieve therapeutic potency and imaging potential [31]. A more comprehensive analysis has recently been conducted by Pellegrino and coworkers discussing the current state of magnetic-based stimuli-responsive systems [32]. Nevertheless, the cytotoxicity of IONP is still debated, with some reports revealing increased toxicity (i.e., disruption of cell cytoskeleton) [33] and others demonstrating no toxicity (i.e., no increase of reactive oxygen species) [34]. Additionally, although IONP were successful in generating initial buzz under familiar names such as Feridex, they ultimately failed commercially due to adverse side effects and lack of diagnostic utility [35]. Despite this, a resurgence of their use has been found in the treatment of iron deficiency with more recent efforts exploiting the use of ferromagnetic IONP (i.e., permanent magnetism) for diagnostic imaging, thereby reaffirming the multitude of applications possible with IONP.

### 2.2. Gold Nanoparticles

Similar to IONP, gold-based nanoparticles have also gained significant popularity over the past decade, seeing applications ranging from optical bioimaging to detection of cancer. Features such as high surface area to volume ratio coupled with cytocompatibility and stability have made gold an ideal candidate for photothermal therapy [36]. In addition to these properties, ease of synthesis and conversion of heat using near-infrared (NIR) light have enabled the use of gold (e.g., nanoshells, nanorods, hollow gold) [37] for a variety of photo-triggered treatments. Specifically, using surface plasmon resonance for photodynamic therapy has drawn particular interest. In particular, exploitation of the combined resonant oscillation of free electrons present on the particle surface, thereby outputting a sharp absorption band, has led to the use of gold nanoparticles in a variety of imaging and therapeutic applications [38]. More so, the ability to conjugate antibodies onto the nanoparticle surface paved the way for direct electron microscopic visualization while minimal toxicity and light scattering efficiency opened the door for a multitude of biomedical applications. Khlebtsov et al. have shown promising results of multifunctional nanoparticles consisting of gold-loaded hematoporphyrin-doped silica particles as an antimicrobial therapeutic [39]. Others have also shown promising applications of gold-based nanoparticles as antibiotic [40] and vaccine [41] delivery systems.

Despite numerous advantageous features, concern over their cytotoxicity still remains. A study designed to evaluate the cytotoxic effects by Soenen et al. [42] revealed that high concentrations (200 nM) led to the formation of reactive oxygen species, resulting in a 20% decrease in cell viability after 24 h. Nevertheless, the same study exhibited that a concentration of 100 nM showed negligible toxicity. To mitigate toxicity, Choi et al. [43] designed a gold-loaded nanocarrier that was shown to increase circulation time and tumor accumulation while minimizing disruption of metabolic activity and cell viability. The ability to localize more gold to the tumor site through an increase in circulation enables a hyperthermia-based approach to be more effective and reveals a promising tool for translation into the clinic.

Although great success has been observed with gold nanoparticles using in vitro and in vivo models, lack of homogeneity in human cancer prevents gold from showing the same success in the clinic. In addition, the high cost associated with development of gold nanoparticles remains as another barrier preventing clinical translation [35,44]. For this reason, continued investigation in the scale-up for commercialization and clinical trials needs to be re-evaluated and optimized to meet the demand of the clinic. However, as is often the case with nanoparticles, delineation of nanoparticle accumulation at the target site can often be difficult to assess in clinical trials, serving as a barrier to their proper investigation.

### 2.3. Quantum Dots

Showing similar rise in popularity are non-metal theranostics such as quantum dots (QD), colloidal particles that can range in size from 1 to 10 nm in diameter [45,46]. These semiconductor nanocrystals, synthesized using a cadmium selenide (CdSe) core with a zinc sulfide layer to maintain desirable crystallinity and homogeneity, are able to emit light and exhibit distinctive optical qualities that are not found in organic dyes or florescent probes [47]. These qualities include exhibiting high luminescence, a more stable and restricted emission spectrum, and a broader excitation field [45,48]. This is helpful in monitoring long-term studies such as the interactions of multi-labeled biological markers in cells. Additionally, the ability to fine-tune the fluorescence emission of QD from ultraviolet to near-infrared wavelengths has exhibited beneficial properties for studying the extravasation of cancer cells in vivo. For example, conjugating antibodies that target different tumor markers onto QD allows for the real-time imaging of cancer cells as they metastasize [47].

Additionally, surface modification of QD can provide further benefits. To create water-stabilized QD with increased photostability and enhanced functionality, Medintz et al. were able to use ligand exchange to replace hydrophobic capping ligands with hydrophilic bifunctional ligands [49]. These aqueous QD can be used for fluorescence imaging or to trace receptor mediated trafficking in live cells and for long term labeling of endosomes without any drastic harmful effects [50]. After successful in vitro studies, Gao et al. developed a copolymer coated QD to target and image prostate cancer in vivo [51]. Using this method, the tumor could be actively probed by the antibody conjugated QDs and imaged in live animals. Further tuning the size to favor rapid clearance from the body and applications calling for high sensitivity have the potential to make QD an integral part of imaging the human body.

**Figure 2:**
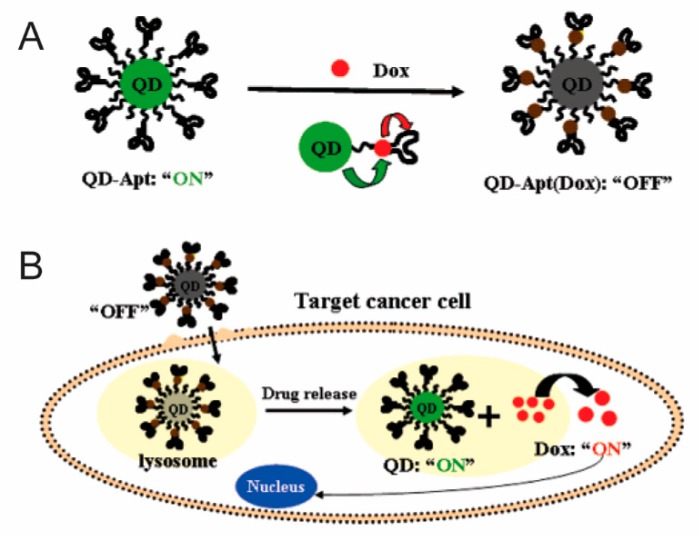
(**A**) Schematic illustration demonstrating the Bi-FRET-based QD-aptamer-doxorubicin nanoparticle. This approach results in the simultaneous quenching of QD and doxorubicin; QD fluorescence is quenched by doxorubicin while doxorubicin fluorescence is quenched by QD; (**B**) Schematic illustration depicting the internalization via the PSMA endocytosis pathway. Internalization results in the release of doxorubicin from the conjugated nanoparticle, thereby resulting in cell death and the triggering of QD fluorescence. Images reproduced from [53], with permission from American Chemical Society, 2007.

Nevertheless, caution must be taken when using QD in vivo. Many studies indicate that the use of cadmium is toxic and that it possesses DNA-damaging properties. Other groups suggest that the use of cadmium in the cellular environment also results in the formation of reactive oxygen species that contributes to cell death. Thus to prevent or reduce these harmful effects, passivation can be used to protect the core from oxidation and lower the toxic effects [52]. Nevertheless, more recent efforts have aimed to harness the diagnostic potential of QD and couple them with a chemotherapeutic such as doxorubicin, a commonly used anthracycline drug. In a study performed by Bagalkot et al., QD were used to develop a QD-aptamer-doxorubicin conjugate capable of targeting cancer cells (Figure 2) [53]. This approach harnesses the targeting potential of the aptamer specifically selected to localize at prostate cancer cells expressing the antigen. Following binding to the target, the conjugated doxorubicin is released, resulting in the activation of the QD core, consequently allowing for the simultaneous imaging of the cancer cells. However, to be properly translated into the clinic, significant work still needs to be performed investigating the toxicity of QD. As it currently stands, QD translation into in vivo models often portrays difficulty in identifying the dominant and compensation mechanism employed [54], spurring a need for a multi-modal QD system. In addition, further evaluation of toxicity is needed before QD can reach clinical translation status.

## 3. Multistage Nanovectors

Despite all the advantages first-generation nanoparticles provide [55,56], the many biological obstacles they are required to overcome have led to the development of several delivery vectors designed to decouple the multitude of tasks required to bypass these barriers [57,58,59]. Previously, our group introduced multistage nanovectors (MSV) [60,61], engineered to systemically shield, transport and reliably deliver therapeutic and imaging agents, thereby making them ideal for theranostics applications [62,63]. Designed using porous silicon due to its biocompatibility and degradability [64,65], well-established fabrication techniques make it possible to uniquely control parameters such as shape, size, and porosity that can aid in the strategic negotiation of biological barriers [56,57,66]. As one example, mathematical modeling has revealed that MSV exhibit superior margination and adhesion during systemic circulation, favoring the release of a payload into the extracellular space [67,68]. In addition, functionalization of the MSV surface with biological moieties (e.g., antibodies, aptamers, phages) can further aid in the negotiation of biological barriers such as avoidance of MPS and targeting of inflamed vasculature [8,69]. This versatility, combined with the ability to control the release kinetics of a payload [70], makes MSV a promising tool for theranostics applications [71,72]. Furthermore, porous silicon as a material has been extensively studied for various medical applications including diagnostics, drug delivery, implantables, and tissue engineering [73,74].

### Nanoparticle Loading into Multistage Nanovectors

The nano-sized pores of MSV facilitate the loading and retention of several types of nanoparticles that effectively bestow MSV with novel therapeutic and diagnostic functions [75]. For example, loading MSV with liposomes containing small interfering RNA (siRNA) directed against the EphA2 oncoprotein resulted in the sustained delivery of siRNA and silencing of the protein in ovarian tumors for up to three weeks, substantially extending the silencing impact of free liposomes that previously required biweekly administration to achieve a similar response [76]. This work was further expanded to demonstrate an enhanced tumor response by combining chemotherapy (e.g., Paclitaxel and Docetaxel) with sustained EphA2 siRNA delivery using MSV [77]. This approach resulted in a significant reduction in tumor burden with complete inhibition of tumor growth when combined with chemotherapy in two different tumor models, including a highly aggressive and chemoresistant model (i.e., HeyA8-MDR). In addition, this approach of MSV/siRNA was validated in treating breast cancer by delivering siRNA-targeting ataxia telangiectasia mutated (ATM) genes using liposomes [78] or by modifying the surface of MSV with polyethyleneimine to form nanocomplexes within the pores to deliver ATM [79], STAT3, and GRP78 siRNA [80] inducing significant reduction in cancer stem cells. MSV loading with paclitaxel micelles exhibited a similar sustained delivery and suppressed tumor growth with a single administration, confirming the sustained release characteristics of MSV upon loading with nanoparticles [81].

**Figure 3:**
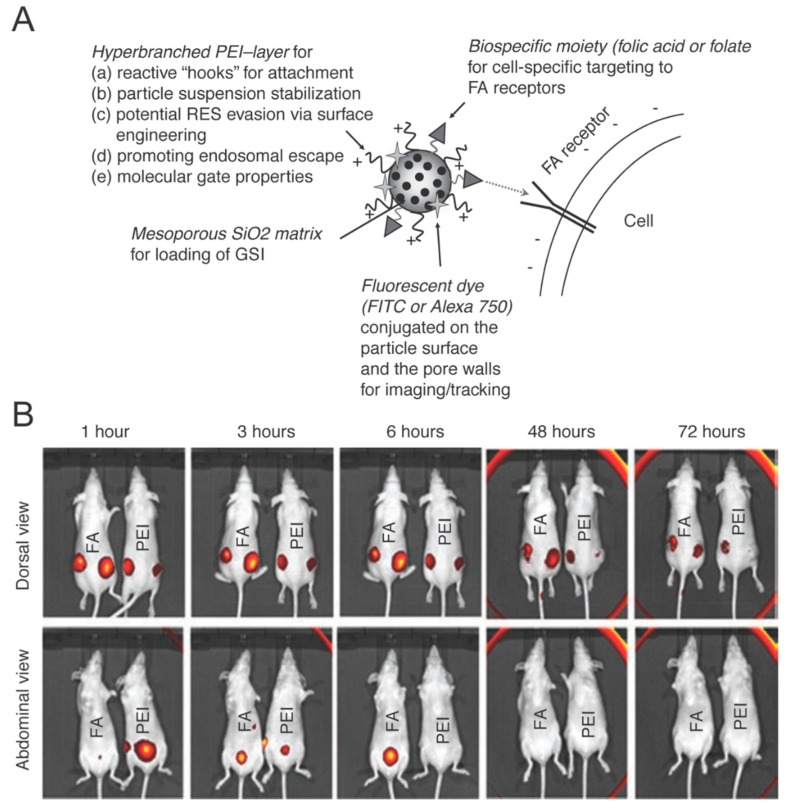
(**A**) Schematic illustration depicting the mesoporous silica nanoparticle matrix functionalized with a folate targeting moieties and fluorescent dyes; (**B**) Dorsal (top) and abdominal (bottom) in vivo images of tumor-bearing mice treated with non-conjugated mesoporous silica nanoparticles (PEI) and folate conjugated nanoparticles (FA) over a 72 h time period. Dorsal images depict nanoparticle accumulation in the tumor while abdominal images depict accumulation in the bladder. Mice were each inoculated with two tumors. Images reproduced from [82], with permission from Cell Press, 2011.

Lastly, a cooperative thermal therapy approach for breast cancer was demonstrated by loading NIR responsive hollow gold nanoparticles into MSV [82]. This approach enabled a two-fold increase in heat generation and more efficient cell killing independent of genetic mutations expressed by the breast cancer cells (i.e., HER2 vs triple-negative) (Figure 3). This cooperative effect was generated due to the collective electromagnetic dipole-dipole coupling of gold nanoparticles within MSV, resulting in a coherent thermal spot-source allowing for more efficient heat dissipation and increased energy transfer and heat production.

The diagnostic potential of MSV was further evaluated by investigating emerging properties upon loading with contrast agents. The loading of MSV with gadolinium-based contrast agents (Magnevist, spherical fullerenes and carbon nanotubes encapsulating gadolinium ions) revealed a 50-fold increase in the relaxivity of MRI compared to clinically available contrast agents and, thus, significantly enhanced the T_1_ contrast possible [83]. This improved relaxivity and contrast enhancement was attributed to geometric confinement of the contrast agents within the pores of MSV. This confining effect resulted in an increased tumbling rate, thus inhibiting the ability of the contrast agents to rotate freely and effectively, reducing the mobility of the water molecules. The impact of confinement was studied by loading Magnevist in MSV with various pore sizes and demonstrated that smaller pores bestowed greater relaxivity enhancement [84].

In addition to gadolinium, MSV loaded with superparamagnetic iron oxide nanoparticles (SPION) demonstrated increased negative contrast suitable for T_2_-weighted MRI compared to free SPION [85]. Furthermore, MSV have been successfully loaded with fluorescent QD [86] and carbon nanotubes [87] with their surface allowing for the covalent attachment of NIR fluorescent dyes, radioactive molecules, and therapeutic agents [88]. The flexible and versatile nature of MSV has the potential to generate theranostic agents by co-loading nanoparticles that individually provide therapeutic (e.g., siRNA, micelles, gold) or diagnostic (e.g., gadofullerenes, gadonanotubes, SPION, QD) action and thus whose combination would result in treatment and imaging. Alternatively, the surface of MSV could be used to attach diagnostic and therapeutic agents, permitting one to use the full porous matrix to load a nanoparticle payload. Furthermore, any current or future theranostic nanoparticle smaller than 100 nm can be incorporated into MSV with relative ease, enabling advanced generations of theranostic agents.

## 4. Bio-Inspired Theranostics

Recently, bio-inspired approaches have gained increasing popularity in overcoming the current limitations of drug delivery systems such as biocompatibility, toxicity, and targeting [89,90]. FDA-approved Abraxane, albumin-bound paclitaxel, represents the first example of a bio-inspired approach and has been shown to improve circulation time while reducing unwanted side effects of chemotherapy. Harnessing albumin’s innate ability to transport hydrophobic molecules and interact with endothelial cells has led to Abraxane exhibiting increased efficacy of paclitaxel, thereby demonstrating itself as an effective adjuvant therapy. This manipulation of biological matter and its incorporation into synthetic carriers and payloads was proposed to both improve the delivery of drugs and assist in accumulation of imaging agents. As such, theranostics based on the mimicry or incorporation of biological components were developed to exploit all levels of biological complexity.

It is therefore not only important to select a material that works compatibly when administered but to also consider rational design when engineering drug delivery vectors. Although nanoparticle design has traditionally centered on the use of spherically-shaped particles due to ease of synthesis, more recent efforts have been biologically inspired, leading to the design of vehicles that are strategically shaped to optimally travel within the blood stream and overcome biological barriers. In the preceding case (Section 3), MSV were designed to mimic the size and shape of red blood cells to increase margination towards vessel walls. Similarly, other efforts have drawn inspiration from bacteria’s worm-like structure (e.g., filomicelles) [91]. Specifically, the elongated shape of filomicelles and nanoworms have shown great promise as delivery vehicles both for chemotherapeutic delivery [92] and imaging applications [93]. In addition, hyper-branched polymeric structures have also been designed to covalently link drug molecules to a substrate, providing controlled drug release mediated through degradable linkages [94]. Nevertheless, although shape has played a pivotal role in drug delivery carrier design, other efforts have leveraged physical incorporation of biological components. As such, this section will highlight some key aspects of bio-inspired theranostics such as enzymatic substrates, natural-derived transporters, viruses, and cells.

### 4.1. Proteases

Proteases (e.g., caspases, metalloproteases (MMP), furin) have been identified as a component of the tumor environment that is commonly overexpressed and, thus, is a prime tool to exploit for the development of bio-inspired strategies. Recently, nanoformulated protease substrates were proposed as a new research tool to investigate proteolytic activity in the intra- and extra-cellular space. These substrates work by taking advantage of the cleavage of monomeric units that polymerize after cleavage, functioning as an enhanced fluorescent signal or theranostic agent [95,96]. Another strategy designed by Kim et al. involved the use of MMP and cathepsin B as an activation mechanism for fluorescent nanoprobes [97]. In this way, the imaging of a tumor area can be enhanced knowing that proteolitic enzymes are readily present in the tumor microenvironment, thereby leading to the cleavage of the imaging probe and higher specificity of imaging agents. Wong et al. developed a QD-loaded gelatin multistage nanoparticle designed to degrade in the presence of MMP-2, a protease highly expressed in the tumor microenvironment, thereby releasing smaller sized QD that readily diffuse into the tumor [98].

Cathepsins, monomeric proteases, have also been identified as viable targets to be employed in targeted-based therapies. Typically activated in low pH environments such as lysosomes, cathepsins have been abundantly expressed in various malignant tumors and are known to increase cancer cell recruitment. In one strategy, PEG was combined with cathepsin B to form a liposomal nanoparticle that facilitates the targeting of cathepsin B expressing cancer cells, allowing the release of a therapeutic payload at a target site [99]. Cathepsin was similarly used in an effort to mitigate the unwanted side effects of chemotherapeutic camptothecin derivatives [100]. When cathepsin B was conjugated onto a camptothecin derivative, similar anti-tumor effects were observed without any toxic effects. This method demonstrates considerable promise in the use of proteases to develop viable bioinspired strategies.

### 4.2. Lipoproteins

Similar to proteases, lipoprotein-based nanoparticles have also been extensively evaluated as a suitable bioinspired approach for the transport of theranostic payloads [101]. This class of nanoparticles is biochemically synthesized by the body and governs the transport of lipids, enabling fats to be carried in the blood stream. Additionally, lipoproteins possess innate biocompatibility properties, inspiring the design of long circulating particles aimed at improving the transport of hydrophobic payloads. Unique properties such as their small size (<40 nm) and amphiphilic nature favor their diffusion, in addition to their payload, deeper into the tumor mass. As such, low-density (LDL) and high-density (HDL) lipoprotein-based carriers have been developed to exploit these properties and increase the delivery of therapeutic and imaging agents through weak chemical interactions (i.e., covalent bonds) and the exchange of a hydrophobic core with a payload of interest.

For example, LDL conjugated with radiolabelled tracers was shown to accumulate in the tumor within 24 h of injection, shedding light to the abnormal traffic of these molecules and lipid metabolism during cancer [102]. Furthermore, LDL has been shown to possess great propensity in accommodating a variety of agents for photodynamic therapy (e.g., NIR-molecules [103,104,105]) and can further be modified to target cancer cells. For example, Zheng et al. showed that by conjugating a tumor-homing molecule through a lysine substitution and coupling LDL with folate, accumulation of LDL in cancer cells is improved [106]. Conversely, HDL-based delivery systems rely on the over expression of their natural receptor, scavenger receptor class B type I, in many cancer cells [107,108]. It was hypothesized that HDL could represent a major source of cholesterol for growing neoplastic lesions [109]. This led to increased interest in HDL-based carriers and the loading of chemotherapeutics (e.g., paclitaxel) [110] and NIR agents capable of generating reactive oxygen species under light irradiation, resulting in the killing of cancer cells.

### 4.3. Viral & Cellular Vesicles

Further inspiration for suitable bioinspired approaches was found by imitating the working mechanism of viruses. Their enhanced ability to target and integrate their genome into the DNA of human cells makes them a promising tool for drug delivery. In particular, adenoviral particles were investigated as ideal carriers for gene therapy in vivo with recent efforts focused on coupling these carriers with metallic particles for improved imaging and curative properties. Specifically, iron particles were shown to readily absorb onto the adenovirus surface resulting in a hybrid particulate with promising theranostic properties. To further refine and standardize the hybridization process, Everts et al. modified the surface of adenoviral vectors with gold nanoparticles [111]. This led to the ability to use the adenoviral vector for its tumor-associated antigen homing ability and the gold nanoparticles for their ablation properties.

**Figure 4:**
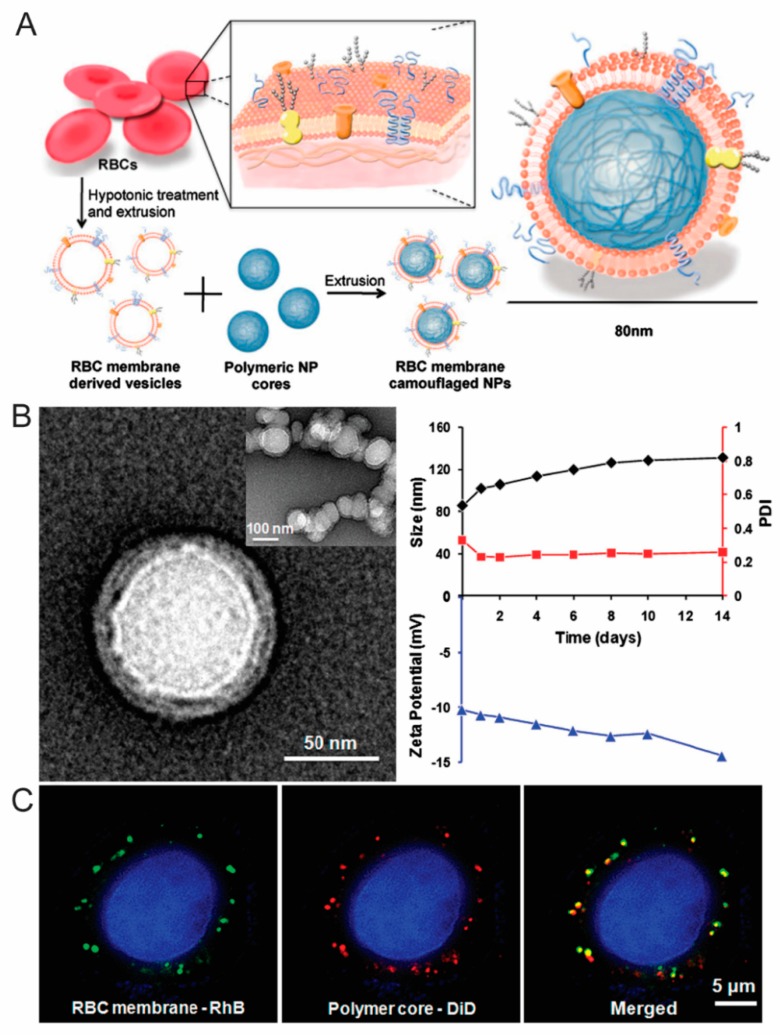
(**A**) Schematic illustration demonstrating the fabrication of erythrocyte-coated PLGA nanoparticles; (**B**) Transmission electron microscope images of erythrocyte-functionalized PLGA nanoparticles and DLS measurements depicted nanoparticle size (black), PDI (red), and zeta potential over 14 d; (**C**) Fluorescent microscope images depicting the colocalization of erythrocyte membranes (green) and PLGA cores (red) following internalization by cervical cancer HeLa cells after 6 h. Images reproduced from [114], with permission from National Academy of Sciences, 2011.

Conversely, inspiration drawn directly from cells found in the body (i.e., erythrocytes, leukocytes, mesenchymal stem cells) have also gained increasing prominence. Over the past two decades, erythrocytes have been investigated for their biocompatibility, prolonged circulation, and their desirable isolation and manipulation properties. In addition, the ability to load a payload into the cellular body through concentration gradients makes erythrocytes a promising carrier. Methotrexate, a chemotherapeutic used to treat inflammatory diseases, loaded into erythrocytes, represents one of the first examples to successfully inhibit cancer growth. The loading of photo-triggered hematoporphyrin derivatives into erythrocytes has also been shown to provide antibody-mediated delivery of the derivatives with increased efficacy [112]. For prolonged circulation and decreased clearance of IONP, Markov et al. designed a protocol that incorporates IONP into erythrocytes that demonstrated considerable improvements in imaging properties of IONP for MRI [113]. In a similar strategy, Hu et al. incorporated erythrocyte cellular membrane to coat poly (lactic-co-glycolic acid) (PLGA) particles (Figure 4) [114]. Following functionalization with an erythrocyte shell, it was reported that PLGA particles remained in circulation for three days following administration in vivo, demonstrating promising potential as a delivery vector. This approach was later further optimized to combine a hybrid erythrocyte/platelet-derived membrane to provide increased circulation and marry the two distinct functions of each donor cell source [115].

Similarly, cell-derived vesicles known as exosomes have also garnered significant interest due to their small size and protein function. As such, exosomes have been reported as vesicles that facilitate transport of biological materials (e.g., proteins, mRNA) to different tissues by utilizing vascular systems [116]. This has led to attempts to isolate exosomes and load them with therapeutic payloads (e.g., siRNA) to exploit their natural tropism, in addition to their biocompatibility and prolonged circulation, thereby making them a promising tool for theranostics [117]. Despite this, exosomes still lack many of the proteins needed for targeting cancer and overcoming biological barriers to actively target inflammation. Leukocytes, on the other hand, are decorated with many essential proteins needed for bypassing the MPS, communicating with the endothelial layer, and reaching an inflammatory site.

Mesenchymal stem cells (MSC), often favored due to their innate ability to home to inflammation, have also been considering as a unique tool for drug delivery and as a theranostic system. When previously doped with hyaluronic acid, MSC displayed a substantial increase in homing to inflammation when evaluated in vivo using an inflamed ear animal model [118]. To exploit the innate homing observed with MSC, our group functionalized MSV with a photosensitizer and allowed MSC to internalize our nanoparticles [119]. In a breast cancer animal model, MSC demonstrated successful homing to the tumor, thereby facilitating precise photodynamic therapy using a low power laser source. This method resulted in a 70% decrease in tumor cell viability following photodynamic activation, demonstrating cell-based drug delivery as a versatile therapeutic strategy.

Inspired by the innate biological properties of leukocytes, our group developed a tool designed to mimic leukocytes while exploiting the MSV as our foundation. By coating MSV with freshly isolated leukocyte membranes, our group was able to prolong circulation, avoid MPS uptake, and communicate with the endothelium through critical surface markers [72]. Specifically, it was demonstrated that over 150 transmembrane proteins were successfully grafted onto the MSV particle [120] while still maintaining the bioactivity necessary to facilitate vascular permeability [121]. In addition, it was demonstrated that when MSV were functionalized with cellular membrane derived from a syngeneic cell source, prolonged circulation was achieved with a delay in sequestration in vivo [122].

**Figure 5:**
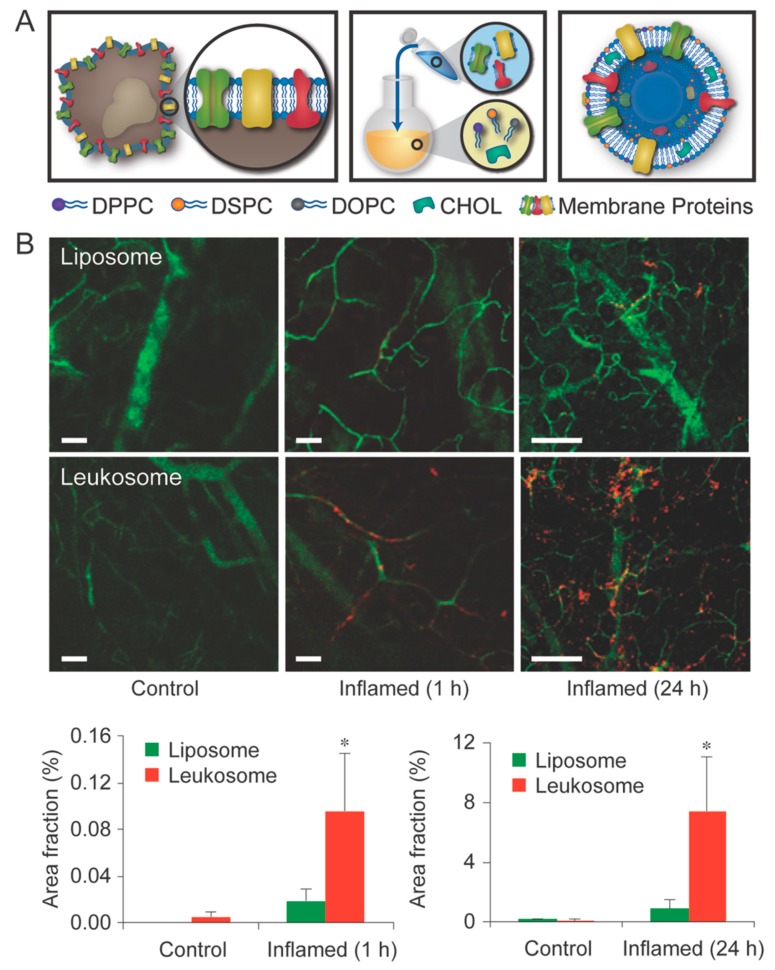
(**A**) Schematic illustration representing the synthesis and formulation of leukosome nanoparticles; (**B**) Intravital microscope images comparing liposome and leukosome accumulation in a lipopolysaccharide-inflamed mouse ear at 1 h and 24 h. Quantitative analysis was performed by calculating the area fraction covered by nanoparticles. Error bars represent the mean ± SD of a minimum of ten fields of view from three mice. Images reproduced from [123], with permission from Springer Nature, 2016.

More recent efforts have incorporated leukocyte proteins directly into a proteolipid formulation, resulting in proteoliposomal vesicles dubbed leukosomes [123]. In this approach, the targeting potential and extended circulation of leukocytes can be granted to all classes of drugs capable of being loaded into liposomal core or within the liposomal bilayer (i.e., hydrophobic, hydrophilic, and amphiphilic). Using leukosomes, a 5-fold increase in targeting inflamed vasculature was displayed when compared to liposomes in as little as 1 h following intravenous administration, with an 8-fold increase being observed at 24 h (Figure 5). These features ultimately allow for greater accumulation at the inflammation site with minimal cytotoxicity to healthy cells, making them promising tools for further evaluation for theranostic-based therapy. Further evaluation of this bioinspired tool revealed a 16-fold increase in breast cancer accumulation relative to liposomes with similar significance also observed in an atherosclerotic plaque animal model [124]. In an effort to evaluate the potential imaging applications using MRI, leukosome bilayers were functionalized with gadolidium chelating phospholipids. This revealed a linear increase in contrast as the leukosome concentration increased, representing promise as an imaging modality and theranostic tool.

As with many clinical therapeutics, ease of translation and scalability remains a valid concern. As such, our group exhibited development of leukosome particles using a commercially available microfluidic system did not hinder production and provided similar grafting compared to traditional thin layer evaporation [125]. Specifically, we demonstrated a comparable transfer of proteins onto the liposomal surface with more efficiency in protein integration observed (i.e., 90% protein integration). In addition, leukosomes were found to remain stable up to one month following fabrication, highlighting the validity of the micro-fluidic system in nanoparticle generation. Particularly, the use of the NanoAssemblr micro-fluidic system was showcased as a promising tool to be used in the fabrication of biomimetic nanoparticles up to 5 mL with scalability to larger micro-fluidic systems (i.e., 1 L batches) made possible with relative ease. Although further studies are still needed, bioinspired theranostics have displayed great promise as therapeutic and diagnostic tools, supplementing an already vast arsenal.

## 5. Conclusions and Future Perspectives

Over the past several years, nanotechnology has spurred the development of a multitude of delivery vehicles and the exploration of a variety of imaging modalities. Theranostics have recently been introduced as a means to unify the dual-step process typically required to diagnose and treat disease. Through the development of one-step theranostic platforms, it is now possible to visualize the disease while simultaneously providing therapy, allowing for the ability to tailor a therapeutic regimen to accommodate the adaptations of the disease and minimize toxicity to healthy tissue. Herein, we briefly highlighted how inorganic nanoparticles have been employed in the use of theranostic-based application (for further reading on the subject see [126,127]). However, to further maximize the efficiency of these theranostic platforms, it is critical to incorporate bioinspired approaches that can be strategically optimized to provide even greater targeting potential and accumulation of a payload. As mentioned in this review, bioinspired approaches have been created to not only harness the innate properties typically presented by the cells of the body, but to offer unique approaches to delivery therapeutic cargoes that display hydrophobic characteristics such as in the case of Abraxane.

In addition, although this review has focused primarily on a select number of nanotechnologies, it is important to note that other materials have also shown promising results as theranostic and biomimetic systems. For example, graphene, carbon nanotubes, and polymeric nanoparticles have garnered significant interest from the scientific community, with trends on social media also highlighting their popularity [128]. In the case of graphene, much work has been performed showcasing the photothermal abilities along with various targeted delivery strategies [129,130]. In addition, the use of other inorganic nanoparticles (e.g., halloysites) have also seen promising use in the stabilization of otherwise agglomerate-prone nanoparticles [131,132]. Overall, with the convergence of theranostic technologies and bioinspired approaches, a new wave of one-step solutions that offer personalized and precision-based technologies can be realized.

Nevertheless, to effectively translate current biomimetic theranostics into the clinic, further investigation into several components is still needed. First and foremost, the issue of scalability remains the primary barrier for translation into the clinic. As the incorporation of biological matter into nanoparticles requires refined and intricate decoration, scalability may not always be a case of simply doubling the materials required for fabrication. In addition, an issue that currently plagues nanoparticle success is lack of homogeneity between patient to patient, opposite of what is commonly observed in small animal models. This is further complicated by the observation of phenomena which are observed in animal models (e.g., enhanced permeability and retention) yet lack sufficient proof in humans. Although significant strides have been made in the translation of the various nanoparticles discussed into preclinical and clinical trials [35], additional work is still needed. Specifically, it is imperative that to effectively continue investigation, careful selection and examination of animal models is employed.
